# Evaluation of carbapenem use in a tertiary hospital: antimicrobial stewardship urgently needed

**DOI:** 10.1186/s13756-018-0449-3

**Published:** 2019-01-07

**Authors:** Di Zhang, Kai Cui, Wei Lu, Hehe Bai, Yuyao Zhai, Sasa Hu, Hao Li, Haiyan Dong, Weiyi Feng, Yalin Dong

**Affiliations:** 1 Department of Pharmacy, The First Affiliated Hospital of Xi’an Jiaotong University, Yanta Western Road No. 277, Xi’an, 710061 China; 2 Department of Management of the Economy, Xi’an University of Posts and Telecommunications, Xi’an, 710061 China; 3 Department of Pharmacy, Taihe Hospital, Hubei University of Medicine, Hubei, 442000 China; 4 Department of Pharmacy, Xi’an Central Hospital, Xi’an, 710003 China; 5 Department of Pharmacy, Xi’an No.4 Hospital, Xi’an, 710004 China; 6 Central Intensive Care Unit, the First Affiliated Hospital of Xi’an Jiaotong University, Xi’an, 710061 China

**Keywords:** China, Antimicrobial stewardship, Carbapenem, Rationality, Carbapenem-resistant *Pseudomonas aeruginosa*

## Abstract

**Background:**

China launched a 3-year rectification scheme for the clinical use of antibiotics in 2011, and a specific scheme for carbapenem use in 2017. The aim of this study was to investigate the effects of government policies on carbapenem use, and their correlation with carbapenem-resistant *Pseudomonas aeruginosa* (CRPA).

**Methods:**

The study was divided into four stages: preintervention (2010), antimicrobial programme (2011–2013), post-antimicrobial programme (2014–2016) and carbapenem programme (2017). A point-score system was proposed for evaluating the rationality of carbapenem use, and evaluated based on the indications, microbial culture, single dose, interval, and duration. Any prescription without a global score of 10 points was judged as irrational. The trend was analyzed by regression analysis, and Spearman correlation analysis was used for testing the correlation.

**Findings:**

The rate of rational use of carbapenems was 29.7% in 2010, and increased by 55.5, 45.2, and 51.5% during the subsequent three stages. The rationality declined slightly during the post-antimicrobial programme (2014–2016) while the consumption of carbapenems was markedly increased. These two parameters improved slightly in 2017. Moreover, the prevalence of CRPA was significantly negatively correlated with the rate of rational carbapenem use (Coefficient = − 0.553, *P* < 0.05), and not with the consumption of carbapenems (*P* > 0.05).

**Conclusions:**

The rational application of carbapenems was related to government policies in this study, with irrational carbapenem use possibly related to the development of CRPA. The current point-score system could be a useful tool for performing assessments.

## Background

Antimicrobial resistance, which is one of the most serious threats to public health globally [1], reduces the available treatment options and increases morbidity, mortality, and costs [2]. The overuse and misuse of antibiotics are crucial factors contributing to the emergence and spread of resistant microorganisms. The WHO reported a programme entitled “Against drug resistance: no action today, no drugs available tomorrow” in April 2011. Meanwhile, the National Health and Family Planning Commission of the People’s Republic of China (NHFPC) launched a special 3-year rectification scheme for the clinical use of antibiotics [3–5]. The government policies were formulated to strengthen the management of antibiotics in clinical applications by setting targets for restricting the kinds of antibiotics and antibiotic prescriptions (the policies are described in detail in the section on methods of antimicrobial stewardship programme). The total antibiotic usage rates in Chinese hospitals have trended downward since then, with the data decreased from 67.3 to 36.8% and from 19.4 to 8.1% for hospitalized patients and outpatients, respectively (2010 to 2017). Meanwhile, the consumption of antibiotics, expressed as the antibiotic use density [6], decreased from 776 defined daily doses (DDDs) per 1000 patient-days (PDs) in 2010 to 457 DDDs/1000 PDs in 2017 [7].

However, the consumption of carbapenems showed a worrying increase [8, 9]. Carbapenems are an important class of antibiotic used for serious infections, and they are characterized by their broad antibiotic spectrum and powerful antibiotic action [10]. Some studies found the consumption of carbapenems was connected with the prevalence of Carbapenem-resistant Gram-negative bacterial pathogens [11, 12], particularly in carbapenem-resistant *Pseudomonas aeruginosa* (CRPA) [13–15]. In March 2017 the NHFPC launched a special programme on the clinical application of carbapenems [16].

The aim of this study was to investigate the effects of government policies on carbapenem use. The appropriate application of carbapenems was analyzed at the First Affiliated Hospital of Xi’an Jiaotong University (FAHXJU). A point-score system was established for evaluating the rationality of carbapenem use, and the correlation with CRPA was analyzed.

## Methods

### Study setting and patient population

This retrospective study was conducted in the FAHXJU, which is a general 2560-bed tertiary-care teaching hospital located in the northwest region of China. This hospital includes all major departments and services, including gynecology and obstetrics, hematology and oncology, cardiovascular, nephrology, infectious diseases, urology, general surgery, pediatrics, and more. A series of antibiotic regulations were implemented in this hospital in July 2011. This study identified all of the hospitalized patients aged ≥18 years who received carbapenems (meropenem or imipenem) during the second weeks of September and December from 2010 to 2017 and were in hospital for at least 3 days. The retrospective design of this study meant that the need to obtain informed consents was waived by the ethics review board.

### Establishing criteria

Based on published previously guidelines on antibiotic use in clinical practice [3, 17, 18] and some references [19–21], we established a point-score system for the evaluation was based on indications, microbial culture, single dose, interval, and duration (Table 1). The point-score system was developed by one specialist in infectious diseases and two pharmacists in a consensus meeting. The system provides a maximum score of 10 points, and assigns a relative weight to each of the items evaluated based on adequacy, efficiency, and safety.

**Table 1:** Score for evaluating carbapenem adequacy

Feature	Question	Answer	Points
Indication^a^	Did the patient need carbapenem administration?	Yes No	5 0
Microbial Culture	Have antibacterial susceptibility tests been done before the use of carbapenems?	Yes No	2 0
Single dosage^b^	Was the dosage correct according to the Chinese labeling and some references [17, 19, 21]?	Yes No	1 0
Interval^b^	Was the interval of carbapenem administration correct according to the Chinese labeling and some references [17, 19, 21]?	Yes No	1 0
Duration^c^	Was the duration of therapy correct according to some references [17–21]?	Yes No	1 0
Total score			0–10

^a^Carbapenems should be administered to infectious patients with severe sepsis or positive microbial cultures that are (not only) susceptible to carbapenems, or who failure to respond to a broad-spectrum therapy such as piperacillin and tazobactam, or cefoperazone and sulbactam. ^b^ Regimen and dosage of meropenem: Renally adjusted dose recommendations are based on doses of 0.5 to 2 g every 8 h. eGFR (estimated glomerular filtration rate, ml·min^− 1^) > 50, no dosage adjustment necessary; eGFR 26 to 50, administer recommended dose based on indication every 12 h; eGFR 10 to 25, administer one-half recommended dose based on indication every 12 h; eGFR < 10 (or intermittent hemodialysis, or peritoneal dialysis), administer one-half recommended dose based on indication every 24 h; continuous renal replacement therapy, 0.5 g every 8 h or 1 g every 8 to 12 h. Regimen and dosage of imipenem: eGFR > 70, 0.25 to 1 g every 6 h or 0.5 to 1 g every 8 to 12 h; eGFR 41 to 70, 0.25 to 0.5 g every 6 to 8 h or 0.75 g every 8 h; eGFR 21 to 40, 0.25 g every 6 to 12 h or 0.5 g every 6 to 8 h; eGFR 6 to 20 (or intermittent hemodialysis), 0.25 to 0.5 g every 12 h; continuous renal replacement therapy, 0.25 g every 6 h or 0.5 g every 6 to 8 h. ^c^ The duration would not be rational without an appropriate indication, or if this was shorter than 3 days and without an adequate reason

A greater impact (0 or 5 points) was assigned to appropriate indications that could be major influences. Carbapenems should be administered to infectious patients with severe sepsis or positive microbial cultures that are (not only) susceptible to carbapenems, or who fail to respond to a broad-spectrum therapy such as piperacillin and tazobactam, or cefoperazone and sulbactam. Microbial culturing should be performed in advance of carbapenem treatment (0 or 2 points). Other mistakes, such as an incorrect single dose, inappropriate interval, or improper duration were given a smaller impact (0 or 1 point). The duration would not be rational without an appropriate indication, or if this was shorter than 3 days and without an adequate reason. Any prescription with a global score other than 10 points was judged as inappropriate.

### Antimicrobial stewardship programme

The nationwide campaign of antibiotic use was launched in Chinese hospitals, with the official document first setting specific targets for antibiotic prescription in 2011 [3]. Antibiotic procurement was restricted to 50 agents in a tertiary hospital, with no more than 3 types of carbapenems. Only meropenem and imipenem were procured in the FAHXJU during the present study period. This document classified carbapenems as specialist antibiotics, and only physicians with senior specialized technical qualifications could prescribe them. Meanwhile, the targets for antibiotic prescription were set at < 60 and < 20% of all prescriptions for hospitalized patients and outpatients, respectively. The submission rate of microbiological specimens should be < 80% when using carbapenems, and the consumption of antibiotics should be limited to ≤400 DDDs/1000 PDs.

During the 3 years from 2011 to 2013, the Chinese government performed nationwide checks on the clinical application of antibiotics. If these indexes did not reach their specified targets, the director of the hospital would be removed. In 2017 the NHFPC launched the special scheme for the clinical application of carbapenems. Since then, the following information should be reported to the provincial health department every month: which kinds of carbapenems were used, and how many of them. Therefore, the study was divided into four stages: preintervention (2010), antimicrobial programme (2011–2013), post-antimicrobial programme (2014–2016) and carbapenem programme (2017).

### Data collection and analysis

The data were collected from the electronic medical recordings in the FAHXJU. The costs were recorded in Chinese yuan and then converted into US dollars (at an exchange rate of 6.4 yuan = US$ 1). Both of the prevalence of CRPA and the consumption of carbapenems were calculated per quarter in the second half of the year. The two kinds of information was collected from the patients in the whole hospital, including the ones without evaluation. Moreover, the date came from outpatient clinics or the emergency room were excluded. The trend was analyzed by regression analysis, and Spearman correlation analysis was used for testing the relation. All of the data were analyzed by SPSS software. The quantitative variables, not conform to a normal distribution, were expressed as medians and interquartile ranges, with the nonparametric Mann-Whitney test for comparisons. Qualitative variables are presented with their frequency distributions, which were compared using the χ^2^ test. Probability values of *P* < 0.05 were considered statistically significant.

## Results

### Clinical characteristics and costs

Table 2 shows the detail information of patients receiving carbapenems. 1774 medical records were included. There were no significant differences in both of gender or clinical department during these stages (*P* > 0.05). The patients were younger in 2017 (*P* < 0.05), compared to those in other stages. Compare to 2010, there was higher eGFR (estimated glomerular filtration rate) and more costs (the total drug costs and hospital costs) during the subsequent three stages (*P* < 0.05). The duration of carbapenem use was shorter in 2010.

**Table 2:** Demographic and clinical characteristics of the patients receiving carbapenem treatment

Characteristic	2010	2011–2013	2014–2016	2017
Patients N	209	440	787	338
Male sex N (%)	132 (63.2)	272 (61.8)	285 (36.2)	127 (37.6)
Age (years)	60 (46–72)	58 (45–70)	57 (46–69)	58 (42–67) ^*^
Department of Medicine (%) ^a^	119 (56.9)	242 (55.0)	430 (54.6)	203 (60.1)
duration of stay in the hospital	21 (13–30)	22 (14–33)	18 (11–27) ^*#^	19 (12–28) ^#^
eGFR ^b^ (ml·min^−1^)	91.9 (65.8–105.7)	99.5 ^*^ (75.2–115.2)	101.8^*^ (80.0-118.2)	98.65^*†^ (64.8–113.2)
Carbapenem duration (days)	7 (5–11)	8 (5–12) ^*^	8 (6–12) ^*^	8 (5–12)
Total drug costs ($)	3713.9 (2121.0–6489.0)	5728.9 ^*^ (3104.0–9054.3)	4931.2 ^*^ (2781.1–8900.5)	5315.3 ^*^ (2521.4–10,418.6)
Hospital costs ($)	6254.8 (3424.7–10,819.4)	10,130.9 ^*^ (5391.9–16,961.9)	8929.2 ^*^ (4796.9–17,886.1)	10,427.0 ^*†^ (4752.2–20,489.7)

^*^P < 0.05 when compared with 2010; ^#^*P* < 0.05 when compared with 2011–2013; ^†^*P* < 0.05 when compared with 2014–2016

^a^The clinical departments were simply dichotomized into medicine and surgery

^b^eGFR, estimated glomerular filtration rate. It was calculated by the formula of Chronic Kidney Disease-EPI

### Evaluation of carbapenem therapy

Table 3 presents the adequacy of carbapenem therapy. The rate of 10-point scores was 29.7% in 2010. The rational use of carbapenems increased significantly after the interventions (*P* < 0.05), but still only about 50% of patients received appropriate carbapenem treatments during the subsequent three stages. In 2010, 22.5% of patients, who did not actually have infections, received carbapenems, but this rate improved markedly thereafter (*P* < 0.05). There were also significant improvements in indications, microbial culture, interval and duration after the intervention. Interesting, the rationality of indexes (indications, microbial culture and duration) declined slightly during the 3 years of the post-antimicrobial programme (2014–2016). These results indicated that the improvement in the rational application of carbapenems was related to the implemented government policies.

**Table 3:** Adequacy of carbapenem treatment

Indicator	2010	2011–2013	2014–2016	2017
Rational prescription (10 point scores), N (%)	62 (29.7)	244 (55.5) ^*^	356 (45.2) ^*#^	174 (51.5) ^*^
Rational indication	126 (60.3)	343 (78.0) ^*^	495 (62.9) ^#^	262 (77.5) ^*†^
Infectious patients	162 (77.5)	411 (93.4) ^*^	719 (91.4) ^*^	314 (92.9) ^*^
Microbial culture	130 (62.2)	403 (91.6) ^*^	663 (84.2) ^*#^	291 (86.1) ^*#^
Rational single dosage	209 (100)	438 (99.5)	784 (99.6)	338 (100.0)
Rational interval	118 (56.5)	337 (76.6) ^*^	637 (80.9) ^*^	271 (80.2) ^*^
Rational duration	117 (56.0)	325 (73.9) ^*^	482 (61.2) ^#^	245(72.5) ^*†^

^*^P < 0.05 when compared with 2010; ^#^*P* < 0.05 when compared with 2011–2013; ^†^*P* < 0.05 when compared with 2014–2016

### Prevalence of CRPA and correlation

**Fig. 1:**
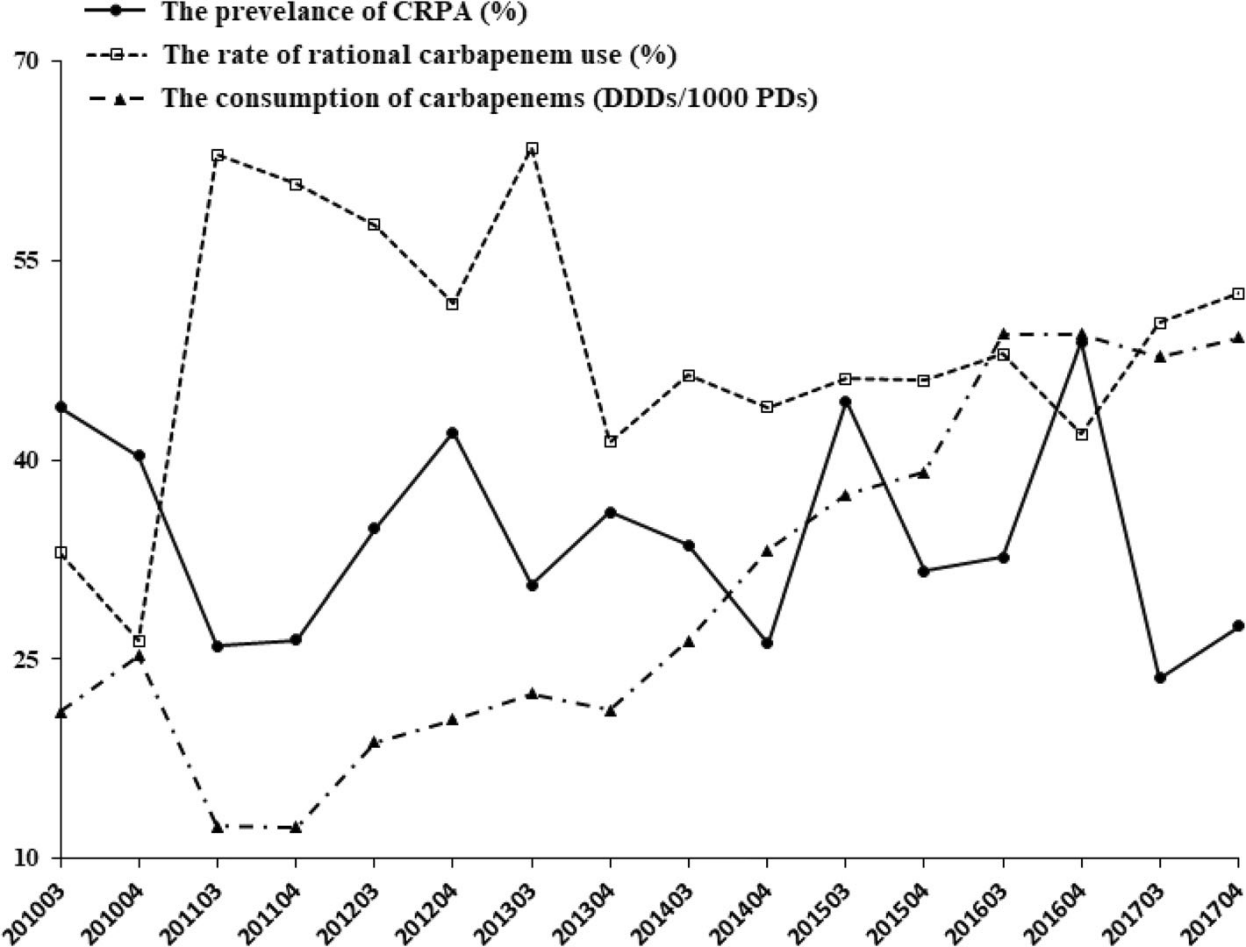
The correlation between the resistant rate of CRPA (carbapenem-resistant *Pseudomonas aeruginosa*) and the rational ratio of carbapenem use (or the consumption of carbapenems) during 2010–2017. The consumption of carbapenems was expressed as defined daily doses per 1000 patients per day (DDDs/1000 PDs)

During the study period there was an overall significantly increasing trend in the consumption of carbapenems, from 21.1 DDDs/1000 PDs during the third quarter of 2010 to 49.2 DDDs/1000 PDs during the fourth quarter of 2017 (*P* < 0.05, Fig. 1). Meanwhile, the prevalence of CRPA and the rate of rational carbapenem use did not reach statistical significance (*P* > 0.05, Table 4). Compared to 2010, the consumption of carbapenems decreased during the 3 years of the antimicrobial programme (2011–2013), with a nadir in 2011 and then increasing from 2012. However, the prevalence of CRPA did not show a similar trend (*P* > 0.05). The prevalence of CRPA showed a fluctuating trend, and it was significantly negatively correlated with the rate of rational carbapenem use (*P* < 0.05, Table 4). Therefore, the irrational use of carbapenems might be an important contributor to the development of CRPA.

**Table 4:** The trend and correlation between the prevalence of CRPA and the rate of rational carbapenem use (or the consumption of carbapenems) during 2010–2017

	Trend	Slope (β)	*P*	Correlation with CRPA	
				Coefficient	*P*
The prevalence of CRPA ^a^	Stable	−0.006	0.51	/	/
The rate of rational of carbapenem use	Stable	0.004	0.73	−0.553	0.026^*^
The consumption of carbapenems ^b^	Increasing	0.051	0.00	0.071	0.795

NOTE. All of the data were calculated per quarter in the second half of the year

^a^CRPA, carbapenem-resistant *Pseudomonas aeruginosa*

^b^The consumption of carbapenems was expressed as defined daily doses per 1000 patients per day (DDDs/1000 PDs)

## Discussion

This study explored the effects of government policies on carbapenem use in a tertiary hospital in China. It included 1774 patients, comprising 209 patients at preintervention (2010), 440 patients in the antimicrobial programme (2011–2013), 787 patients in the post-antimicrobial programme (2014–2016) and 338 patients in the carbapenem programme (2017).

Our establishment of a simple point-score system revealed opportunities for improvement and provided baseline data before starting an antimicrobial stewardship programme. The system was also found to be a practical tool for assessing the results of interventions. There was a high rate of irrational carbapenem use before an intervention, with less than 30% of the decisions about carbapenem meeting all of the criteria for adequacy in 2010. The rate of rational use increased significantly after implementing the antimicrobial programme, by 55.5, 45.2, and 51.5% during the subsequent three stages. Moreover, the consumption of carbapenems was lowest when the rate of rational use was highest during the antimicrobial programme (2011–2013). The 3-year rectification scheme was considered the strictest programme related to the clinical use of antibiotics in Chinese history, and a law about antibiotic use was enacted in 2012 [22]. However, the rate of rational carbapenem use declined during the post-antimicrobial programme (2014–2016), and the consumption of carbapenems was markedly elevated during that period. The carbapenem programme was launched in 2017 in Chinese hospitals, since when both the rational use and consumption of carbapenems have improved slightly. However, the effect of the carbapenem programme was not ideal due to the lack of a specific index to limit the application of carbapenems.

The main indicator of the point-score system is the indication. Our system employs simple-to-apply criteria, with a score of < 5 points indicating that a lack of rational indication. The rate of irrational indications was almost 40% in 2010, and one of the important causes was carbapenems being administered to patients who did not have infections. The rate of rational indications was < 80% throughout the research period, which represented a major problem related to carbapenem use. Moreover, it should be stressed that the duration of carbapenem use was judged based on the appropriate indication. Therefore, these two parameters showed similar trends. The median duration of carbapenem use was 8 days after the intervention, which was consistent with that found by Gauzit et al. in French hospitals [23]. The submission rate of microbiological specimens and the rational interval were lower in 2010. Erbay et al. suggested that applying susceptibility testing could decrease the risk of the inappropriate use of antibiotics [24]. The application of an antimicrobial programme resulted in significant increases in both the submission rate of microbiological specimens and the rational interval. In contrast to other indicators, the application of a single dosage was always reasonable. Patry et al. also found that only 0.5% of doses were inappropriate [21].

The relationship between carbapenem use and the prevalence of CRPA was also investigated. A particularly interesting finding was that the prevalence of CRPA was significantly negatively correlated with the rate of rational carbapenem use (*P* < 0.05), rather than with carbapenem consumption (*P* > 0.05). Although the causal relationship between antibiotic use and antimicrobial resistance is difficult to quantify due to the various settings and measures studied and the presence of related biases, such a relationship is generally accepted as being present [25]. Some studies have found an increased consumption of carbapenems to be mainly due to the appearance of CRPA [13–15]. However, Tofas et al. found that carbapenem use was not a significant risk factor for the development of CRPA [26], indicating that the appropriate use of carbapenems might be a major contributing factor, which is consistent with the findings of our study. Furthermore, the total drug costs and overall hospital costs were significantly increased, and these factors should be further studied when assessing the reasonableness.

This study was subject to several limitations. We have proposed a point-score system that includes qualitative and quantitative indicators for assessing the adequacy of prescription in a non-biased way. This scoring system was more suitable for management because of the various complexities associated with the clinical application of carbapenems. First, it should be considered whether alternative therapeutic medicines are available when the indication is appropriate. Using narrow-spectrum antibacterial agents might be a better choice. Second, pharmacokinetic and pharmacodynamic parameters should be taken into account. Ikawa et al. [27] found a regimen of 0.25 g every 12 h would be sufficient against high-susceptibility bacteria such as *E. coli*, whereas our scoring system judged this dosage regimen as irrational. Third, other issues should also be considered, such as whether a carbapenem was used with another antibiotic and which kind of antibiotics was combined, or whether antimicrobial therapy was de-escalated. Fourth, this study had a single-centre, retrospective design, and sampling analysis was used. Fifth, the clinical departments were simply dichotomized into medicine and surgery, which did not reflect the complexity of clinical practice. Finally, there might be various reasons for the prevalence of CRPA decreasing. However, the present study was mainly focused on changes in the consumption and rationality of carbapenem use in a hospital.

Despite these limitations, we believe that our study is valuable since we analyzed a large amount of data collected over several years. We were also able to confirm how government policies influence carbapenem use, and demonstrate that the point-score system could be a useful tool for performing assessments.

## Conclusions

The rational use of carbapenems is closely related to government policies in China. More effective and implemented policies related to the application of carbapenems need to be developed. Moreover, the irrational use of carbapenems might be a very important factor underlying the development of CRPA. The point-score system developed in this study is simple to apply, and its utilization could promote the rational use of carbapenems.

## Abbreviations

CRPA: carbapenem-resistant Pseudomonas aeruginosa
DDDs/1000 PDs: defined daily doses per 1000 patient-days
FAHXJU: First Affiliated Hospital of Xi’an Jiaotong University
NHFPC: National Health and Family Planning Commission of the People’s Republic of China
